# Virtual-reality-based social cognition and interaction training for patients with schizophrenia: A preliminary efficacy study

**DOI:** 10.3389/fpsyt.2022.1022278

**Published:** 2022-11-17

**Authors:** Zhi-Hua Shen, Meng-Hui Liu, Yue Wu, Qian-Qian Lin, Yong-Guang Wang

**Affiliations:** ^1^Affiliated Mental Health Center and Hangzhou Seventh People’s Hospital, Zhejiang University School of Medicine, Hangzhou, China; ^2^Fuyang Third People’s Hospital, Hangzhou, China; ^3^The Fifth Hospital of Ruian, Wenzhou, China; ^4^Zhejiang Provincial Institute of Drug Abuse Research, Hangzhou, China

**Keywords:** virtual reality, social cognition and interaction training, schizophrenia, social cognition, social functioning, psychosis

## Abstract

**Background:**

Social cognition and interaction training (SCIT) is a psychosocial intervention program for patients with psychosis, designed to improve their social functioning by improving social cognition. Although the feasibility and efficacy of SCIT have been verified, patients with schizophrenia tend to suffer from motivational deficits and low treatment adherence. It has been suggested that using virtual reality (VR) technology might be effective in addressing these issues. In this study, we aimed to develop a VR-based SCIT and compare its efficacy with that of traditional SCIT.

**Materials and methods:**

We developed a novel VR-based social cognition and interaction training (VR-SCIT) that combines traditional SCIT (TR-SCIT) intervention with VR technology. Participants were randomly assigned in a 1:1:1 ratio to the VR-SCIT (*n* = 28), TR-SCIT (*n* = 30), or waiting-list groups (*n* = 29). All treatments were combined with treatment-as-usual. Assessments of social cognition (i.e., Chinese version of Face-Affective Identification Task, Chinese version of Social Cognition Screening Questionnaire) and social functioning (i.e., Chinese version of Personal and Social Performance Scale) were administered from baseline to post-intervention.

**Results:**

Patients receiving VR-SCIT and TR-SCIT showed a significantly greater improvement on the assessments of emotion perception (Cohen’s *d* was 1.66, 0.55, and 0.10 for VR-SCIT, TR-SCIT, and Waiting-list, respectively), hostile attributional bias (Cohen’s *d* was 0.48, 0.44, and 0.05 for VR-SCIT, TR-SCIT, and Waiting-list, respectively), metacognition (Cohen’s *d* was 1.66, 0.76, and 0.06 for VR-SCIT, TR-SCIT, and waiting-list, respectively), and social functioning (Cohen’s *d* was 1.09, 0.90, and 0.20 for VR-SCIT, TR-SCIT, and waiting-list, respectively) from baseline to post-intervention, compared to those in waiting-list group. Additionally, VR-SCIT showed an advantage over TR-SCIT in improving emotion perception and metacognition with higher treatment compliance.

**Conclusion:**

These preliminary findings indicate that VR-SCIT is a feasible and promising method for improving social cognition and social functioning in patients with schizophrenia.

## Introduction

Schizophrenia is a chronic, severe, and crippling mental illness, with a lifetime prevalence of 0.6% in China, which results in a substantial societal burden (1). Social dysfunction is a core clinical feature of schizophrenia and is associated with poor quality of life (2). Reportedly, social dysfunction in patients with schizophrenia is mostly due to an impairment of social cognition (3).

Social cognition refers to understanding the self and others’ mental states and applying this social knowledge to build social behavior (4). Social cognition plays a critical role in daily social interactions and contributes to social success (5). Social cognition is severely impaired in patients with schizophrenia across a variety of domains, such as emotion processing, social perception, theory of mind, and attributional bias (6). Various interventions have been developed over the last few decades to ameliorate social cognition deficits in schizophrenia (7). Among these, social cognition and interaction training (SCIT) has become one of the most widely accepted clinical practices in patients with schizophrenia (8). The feasibility and efficacy of SCIT in schizophrenia have been verified in different clinical settings and cultural backgrounds (9–18).

Motivation deficits and a lack of ecological validity are the main challenges for the implementation of conventional social interventions (19–22). The lack of motivation is a critical factor that contributes to functional disability in schizophrenia, as it discourages an individual from engaging in and adhering to an effective treatment program (23). Meanwhile, as a result of training approaches that may not adequately reflect the complex, interactive, and dynamic nature of real-life social settings, the effects of conventional social interventions on higher-order social cognitive processes and social functioning may be restricted (24). Furthermore, low session adherence has been reported in previous studies on SCIT (25, 26). For example, Dark et al. (25) reported that session adherence was only around 50% on average in their study, which was close to the minimum threshold needed to ensure treatment effectivity (10, 13).

The use of virtual reality (VR) for psychological interventions with gamification-oriented design has been proposed as a possible way to address these issues (27). VR is immersive, interactive, and dynamic, and it elicits psychological reactions that are similar to those occurring in everyday life. Therefore, it is suitable for simulating a range of social situations and accurately portraying their complexity (28). Simultaneously, due to the controllability of VR, situations may be scripted, repeated, modified, and customized, facilitating systematic practice (29). More importantly, gamification has the potential to compensate for motivational impairments using game design elements (30, 31).

Virtual reality interventions have already been utilized successfully in a variety of settings aimed at evaluating and improving symptoms and functional outcomes in schizophrenia (32–36). These studies show that VR can be tolerated by patients with schizophrenia and other psychotic diseases, and it may be useful as a stand-alone or adjunct treatment for a range of medical and psychological therapies (37, 38).

In this study, we aimed to develop an SCIT based on VR and check its efficacy compared to traditional SCIT. It is expected that gamification of VR-SCIT would be associated with a higher feasibility, which may in turn result in a higher adherence to intervention. We also hypothesized that a well-designed VR-SCIT would show an improved efficacy compared with traditional SCIT.

## Materials and methods

### Participants

Eighty-seven participants were diagnosed with schizophrenia based on the Diagnostic and Statistical Manual of Mental Disorders, Fifth Edition criteria (39). Eligibility requirements included the following: (1) 18–50 years old, right-handed, with normal vision and hearing; (2) meeting the diagnostic principles for Andreasen’s remission criteria (40); and (3) only treated with atypical antipsychotics during the study period. Individuals were excluded if they met the criteria for substance use disorder, intellectual disability, head injury, or disease of the central nervous system. Those who had received previous psychological therapy were also excluded. All participants were recruited from the Seventh Hospital of Hangzhou. All participants in this study gave written, informed consent. Written informed consent was also obtained from the individuals for the publication of any potentially identifiable images or data included in this article. This study was approved by the local ethics committee of the Seventh Hospital of Hangzhou (identifier 2019-004).

**FIGURE 1:**
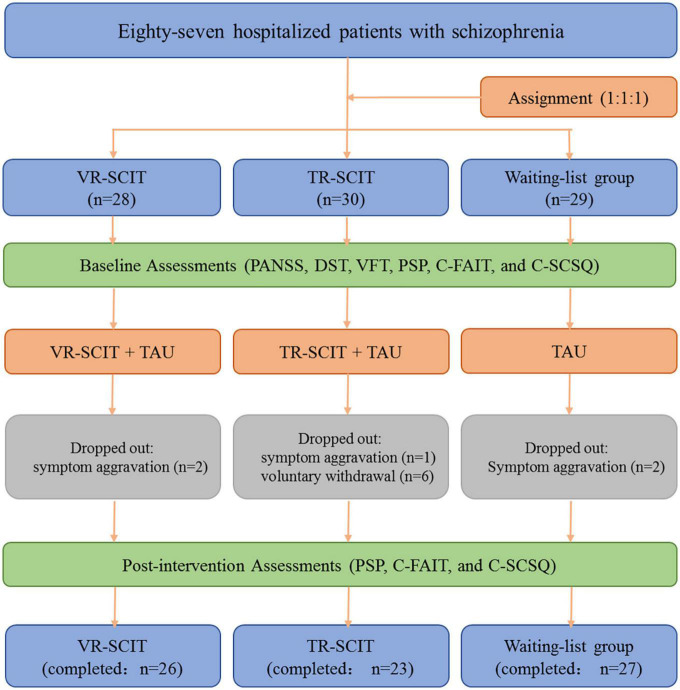
Flowchart of the present work. VR-SCIT, virtual reality based social cognition and interaction training; TR-SCIT, traditional social cognition and interaction training; PANSS, positive and negative syndrome scale; PSP, personal and social performance scale; DST, digit span test; VFT, verbal fluency test; C-FAIT, Chinese version of face affective identification task; C-SCSQ, Chinese social cognition screening questionnaire.

Participants were randomly distributed using a computer-generated list of random numbers and assigned in a 1:1:1 ratio to the following groups: VR-SCIT group (*n* = 28), TR-SCIT group (*n* = 30), or waiting-list group (*n* = 29). These groups received the corresponding treatments (VR-SCIT, TR-SCIT, or no treatment) along with the usual medical treatment. During the study, two patients dropped out for symptom aggravation in the VR-SCIT group, one patient dropped out for symptom aggravation and five for voluntary withdrawal in the TR-SCIT group, and two patients dropped out for symptom aggravation in the waiting list group at week-3 time-point. In addition, one patient dropped out for voluntary withdrawal in the TR-SCIT group at week-5 time-point. Therefore, 26 VR-SCIT, 23 TR-SCIT, and 27 waiting-list participants completed the post-intervention assessments. A flowchart of the study is shown in Figure 1.

### Interventions and procedure

Virtual reality-based social cognition and interaction training was a 3-phase, 10-sessions individual intervention. Each phase took place once a week over a period of 3 weeks. VR-SCIT was delivered to the inpatient department, which was completed by the participants according to the instructions. This involves mass practice and compensatory strategy training (Table 1).

**TABLE 1:** The details of VR-based social cognition and interaction training (VR-SCIT) sessions.

Phases	Goal of the sessions	Gamification in-sessions
Phase-I Introduction and recognize emotions	Session 1: Introduce SCIT and the concept of social cognition	Session 1: Understanding the interplay between emotions, thoughts, and behavior by watching social interaction VR vignettes.
	Session 2: Link the concepts of emotion and mood, and define six basic emotions	Session 2: Facial emotion pictures sorting VR game
	Session 3: Recognize the differences between facial expressions and develop the skills at mimicking the key facial clues	Session 3: Facial emotion Jigsaw Puzzle
	Session 4: Instruct participants on how to estimate confidence in the quality of social judgments	Session 4: Facial emotion recognition VR game with confidence judgments
	Session 5: Improve social cognitive flexibility by using updated information	Session 5: Identifying the dynamically changing facial emotions in the VR vignettes with confidence judgments
Phase-II Figuring out situations	Session 6: Understanding the “Jump to Conclusion”	Session 6: Understanding “Jump to Conclusion” by watching social interaction VR vignettes
	Session 7: Understanding “attributional bias” and think up other guesses	Session 7: VR Interactive Game (i.e., Different attributions lead to different story lines)
	Session 8: Separate facts from guesses	Session 8: Separating facts from guesses based on VR vignettes
	Session 9: Gathering evidence instead of jumping to conclusions	Session 9: Strategy quiz VR game (i.e., Gathering more evidence to narrow it down and get the right answer)
Phase-III Checking it out	Session 10: Practice the learned skills to cope with interpersonal problem in daily life	Session 10: Role-play VR Game (i.e., Participants were required to identify the characters’ facial emotions and to avoid “Jump to Conclusion”)

**FIGURE 2:**
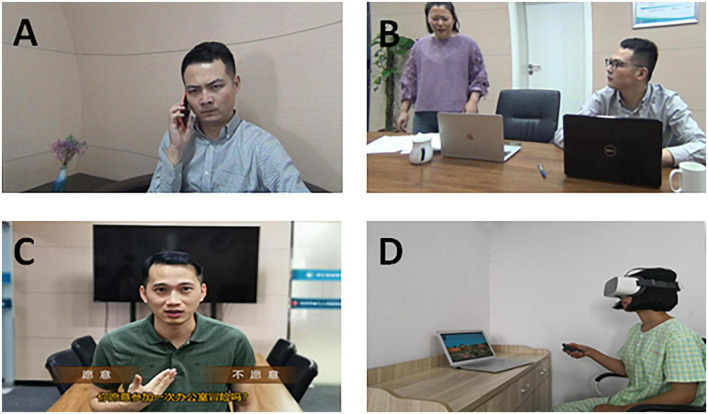
Example of virtual reality based social cognition and interaction training (VR-SCIT) scenarios. **(A)** VR screen shot of phase-I (recognize emotions): A person was answering a phone (voice shielding) with an angry expression on his face, and participants were asked to judge the emotion by recognizing facial cues. **(B)** VR screen shot of phase-II (figuring out situations): After the girl left, another person came in looking for something and messed up her desk. When the girl came back, she blamed the boy for messing up her desk without knowing the facts. **(C)** VR screen shot of phase-III (checking it out): This was a Role-Play Game about office stories, participants needed to make the right choices about identifying emotions, thoughts and behaviors. **(D)** The scene of VR-SCIT intervention.

Phase I consisted of five sessions focused on emotion-perception training. Participants learned about six basic emotions through a picture-sorting game and identified facial cues associated with different emotions through a jigsaw puzzle in VR. Participants practiced several strategies (e.g., mimicking emotions) to aid in emotion recognition, which they were encouraged to try at home. Phase II, with four sessions, addressed theory of mind deficits and attributional bias, in which participants were trained to avoid the pitfalls associated with jumping to conclusions. Participants recognized the harm of jumping to conclusions by viewing scripted social scenarios in the VR environment. Three social strategies can be learned to understand situations: generating perspective-taking, distinguishing social facts from guesses, and gathering additional evidence. Phase III with one session, focused on the integration of learned skills to cope with real-life interpersonal and emotional problems. Participants applied social cognitive skills in a VR role-play game, in which they could consider different possible ways to react to a situation. The game continued until the most appropriate answer was chosen. Participants continued to practice identifying emotions, thoughts, and behaviors, and understanding their interrelatedness (Figure 2).

The TR-SCIT was exactly the same as the previous work we conducted in the community setting [Wang et al. (15)], but with an interval of 2–3 days between sessions. Five SCIT interventions were conducted, each with six inpatient participants. The TR-SCIT took 5 weeks to complete and included three phases: (1) introduction and emotions (seven sessions), (2) figuring out situations (eight sessions), and (3) checking it out (five sessions).

For all participants, antipsychotic medication type and dose were not controlled and instead left to the discretion of the participants’ treating psychiatrists. The psychiatrists who were blinded to the interventional conditions administered all assessments measures to the subjects at both baseline and the endpoint.

### Virtual reality application and virtual reality devices

A Visual Studio code editor was used to write and debug the code. Unity3D software was used to create the UI. Finally, the application runs on the Pico Goblin2 All-In-One (Featuring the Qualcomm^®^ Snapdragon 835 CPU with 4G high-speed LPDDR4-1866 RAM) with the Android 8.1 operating system.

### Assessments

#### Psychopathology and neuropsychological tests

Psychopathology was assessed using the Positive and Negative Syndrome Scale (PANSS) by experienced clinicians (41, 42). The Digit Span Test (DST) and Verbal Fluency Test (VFT) were administered to all participants as neuropsychological background tests (43, 44). The total score on the VFT was the number of animals named correctly in a minute. The total score on the DST was the sum of correctly recalled digits across the digits-forward and digits-backward sub-tests.

#### Social functioning

Social functioning was evaluated using the Chinese version of the Personal and Social Performance Scale (PSP) by psychiatrists who had received training in the use of this method. The PSP total ratings had adequate inter-rater reliability in this study (inter-class correlation coefficient was 0.81) (45).

#### Social cognition

##### Emotion perception

Emotion perception (EP) was assessed using the Chinese version of the Face-Affective Identification Task (C-FAIT) (46). Participants were required to indicate the emotion conveyed in each photograph. The set of C-FAIT test photos used in the current study consisted of 56 photos in total: 48 photos showing six basic emotions (happiness, anger, disgust, sadness, surprise, and fear) and eight photos conveying neutral emotions. The total score for the C-FAIT ranged from 0 to 48, with higher scores indicating better emotion perception.

##### Chinese version of social cognition screening questionnaire

The Chinese version of social cognition screening questionnaire (C-SCSQ) was used to probe the participants’ social cognition, including theory of mind, metacognition, and hostile attributional bias (47). In the C-SCSQ, participants were presented orally with 10 interpersonal vignettes that describe ambiguous interpersonal situations and were required to answer three yes/no questions. In the present work, the scores of theory of mind (ranging from 0–10 to, with higher scores indicating better performance), metacognition (ranging from 7–10 to, higher scores indicate better metacognitive ability), and hostile attributional bias (ranging from 0–5 to, higher scores indicate greater bias) were calculated for each participant.

### Statistical analysis

Pearson’s chi-square test was performed for the female/male ratio and persistence rate of intervention between the groups. One-way analysis of variance (ANOVA) was performed for group comparisons of the demographic and clinical characteristics. A repeated measures ANOVA was conducted for the C-FAIT, C-SCSQ, and PSP scores, with time (baseline vs. post-intervention) as the within-group factor and group (VR-SCIT vs. TR-SCIT vs. waiting-list) as a between-group factor. Within-group effect size estimates were computed using Cohen’s *d* (*d*). *Post-hoc* multiple comparisons were conducted using Bonferroni correction.

## Results

### Demographic and clinical characteristics

Chi-square tests showed no significant difference in sex between the three groups (*χ^2^* = 0.014, *P* = 0.993). No significant difference was found between groups for age (*F*(2,73) = 0.011, *P* = 0.989), education levels (*F*(2,73) = 0.351, *P* = 0.705), DST (*F*(2,73) = 0.034, *P* = 0.966), VFT (*F*(2,73) = 0.020, *P* = 0.980), PANSS-P (*F*(2,73) = 0.005, *P* = 0.995), PANSS-N (*F*(2,73) = 0.071, *P* = 0.932), PANSS-G (*F*(2,73) = 0.203, *P* = 0.817) and PANSS-T (*F*(2,73) = 0.147, *P* = 0.864), respectively. The details are summarized in Table 2.

**TABLE 2:** Demographic and clinical characteristics among groups.

	VR-SCIT group	TR-SCIT group	Waiting-list group	Statistics
	*n* = 26	*n* = 23	*n* = 27	
Sex ration(M:F)	17:09	15:08	18:09	χ^2^ = 0.014, *P* = 0.993
Age (years)	30.81 ± 4.90	31.00 ± 7.48	30.74 ± 6.59	*F*(2,73) = 0.011, *P* = 0.989
Years of education	13.58 ± 2.47	13.22 ± 2.49	13.07 ± 1.73	*F*(2,73) = 0.351, *P* = 0.705
DST	8.15 ± 2.28	8.30 ± 2.01	8.19 ± 2.02	*F*(2,73) = 0.034, *P* = 0.966
VFT	15.69 ± 3.94	15.91 ± 4.13	15.89 ± 4.66	*F*(2,73) = 0.020, *P* = 0.980
**PANSS**				
Positive	15.42 ± 4.27	15.35 ± 3.04	15.33 ± 3.36	*F*(2,73) = 0.005, *P* = 0.995
Negative	16.77 ± 2.93	16.83 ± 3.03	16.52 ± 3.38	*F*(2,73) = 0.071, *P* = 0.932
General	33.00 ± 8.05	34.04 ± 6.97	32.78 ± 7.06	*F*(2,73) = 0.203, *P* = 0.817
Total score	65.19 ± 11.34	66.22 ± 9.42	64.63 ± 10.24	*F*(2,73) = 0.147, *P* = 0.864

Mean ± S.D.; VR-SCIT, virtual reality based social cognition and interaction training; TR-SCIT, traditional social cognition and interaction training; DST, digit span test; VFT, verbal fluency test; PANSS, positive and negative syndrome scale.

### Feasibility

Although there was no significant difference in the overall dropout rate between the VR-SCIT and the TR-SCIT groups (week-3 time-point: 2/28 and 6/30, respectively, *χ^2^* = 1.077, *P* = 0.299; week-5 time-point: 2/28 and 7/30, respectively, *χ^2^* = 1.793, *P* = 0.181), the TR-SCIT group had a significantly higher voluntary withdrawal rate than the VR-SCIT group (week-3 time-point: 5/30 and 0/28, respectively, Fisher’s exact test, *P* = 0.053; week-5 time-point: 6/30 and 0/28, respectively, Fisher’s exact test, *P* = 0.024).

### Social cognition and social functioning

Details of social cognition and social functioning outcomes are summarized in Table 3.

**TABLE 3:** Social cognition and social functioning outcomes among groups.

	VR-SCIT		TR-SCIT		Waiting-list group		Within-group effect size		
	*n* = 26		*n* = 23		*n* = 27				
	Baseline	Post-	Baseline	Post-	Baseline	Post-	VR-SCIT	TR-SCIT	Waiting-list group
	M(SD)	M(SD)	M(SD)	M(SD)	M(SD)	M(SD)			
C-FEIT	30.85(4.17)	37.12(3.34)	31.65(4.91)	34.04(3.64)	30.63(5.51)	31.15(4.82)	*d* = 1.66	*d* = 0.55	*d* = 0.10
**C-SCSQ**									
ToM	6.62(1.20)	7.08(0.94)	6.57(1.20)	7.13(0.87)	6.52(1.31)	6.74(0.91)	*d* = 0.42	*d* = 0.53	*d* = 0.20
HB	1.54(0.91)	1.12(0.82)	1.57(0.99)	1.17(0.83)	1.63(0.79)	1.67(0.78)	*d* = 0.48	*d* = 0.44	*d* = 0.05
MC	8.54(0.65)	9.44(0.41)	8.59(0.68)	9.04(0.48)	8.56(0.73)	8.60(0.70)	*d* = 1.66	*d* = 0.76	*d* = 0.06
PSP	65.77(6.43)	73.08(6.94)	66.30(7.11)	72.39(6.37)	65.56(5.94)	66.67(5.19)	*d* = 1.09	*d* = 0.90	*d* = 0.20

VR-SCIT, virtual reality based social cognition and interaction training; TR-SCIT, traditional social cognition and interaction training; C-FAIT, Chinese version of face affective identification task; C-SCSQ, Chinese social cognition screening questionnaire; ToM, theory of mind; HB, hostile attributional bias; MC, metacognition; PSP, personal and social performance scale.

#### Emotion perception

For C-FAIT, there was a significant main effect of time (*F*(1,73) = 110.619, *P* < 0.001, *partial*η*^2^* = 0.602), indicating that participants had a higher C-FAIT score at post-intervention than at baseline. There was a significant main effect of group (*F*(2,73) = 3.513, *P* = 0.035, *partial*η*^2^* = 0.088) and a significant interaction between group and time (*F*(2,73) = 35.338, *P* < 0.001, *partial*η*^2^* = 0.492). Further analyses revealed that both VR-SCIT (*P* < 0.001) and TR-SCIT (*P* = 0.039) groups had a significantly higher score than the waiting-list group post-intervention, although there was no significant difference among groups at baseline (all *P*≈1.000). In addition, the VR-SCIT group had a significantly higher post-intervention score than the TR-SCIT group (*P* = 0.027).

#### Theory of mind

For theory of mind, there was a significant main effect of time (*F*(1,73) = 11.849, *P* = 0.001, *partial*η*^2^* = 0.140), reflecting that participants had a higher theory of mind score post-intervention than at baseline. There was no significant main effect of group (*F*(2,73) = 0.455, *P* = 0.636, *partial*η*^2^* = 0.012) and no significant interaction between group and time (*F*(2,73) = 0.712, *P* = 0.494, *partial*η*^2^* = 0.019).

#### Hostile attributional bias

For hostile attributional bias, there was a significant main effect of time (*F*(1,73) = 14.957, *P* < 0.001, *partial*η*^2^* = 0.170), reflecting that participants had a lower hostile attributional bias post-intervention than at baseline. There was no significant main effect of group (*F*(1,73) = 1.244, *P* = 0.294, *partial*η*^2^* = 0.033). There was a significant interaction between group and time (*F*(2,73) = 5.099, *P* = 0.008, *partial*η*^2^* = 0.123). Further analyses revealed that the VR-SCIT group had a significantly lower score than the waiting-list group at post-intervention (*P* = 0.047), while no other significant differences were found in the rest of pairwise comparisons at post-intervention or at baseline (*P* ≥ 0.106).

#### Metacognition

For metacognition, there was a significant main effect of time (*F*(1,73) = 82.545, *P* < 0.001, *partial*η*^2^* = 0.531), reflecting that participants had a higher metacognition score post-intervention than at baseline. There was a significant main effect of group (*F*(2,73) = 3.269, *P* = 0.044, *partial*η*^2^* = 0.082) and a significant interaction between group and time (*F*(2,73) = 25.491, *P* < 0.001, *partial*η*^2^* = 0.411). Further analyses revealed that both VR-SCIT (*P* < 0.001) and TR-SCIT (*P* = 0.019) groups had a significantly higher score than the waiting-list group at post-intervention, although there was no significant difference between the groups at baseline (all *P*≈1.000). In addition, the VR-SCIT group had a significantly higher post-intervention score than the TR-SCIT group (*P* = 0.036).

#### Social functioning

For PSP, there was a significant main effect of time (*F*(1,73) = 68.818, *P* < 0.001, *partial*η*^2^* = 0.485), but the analysis did not reveal an effect of group (*F*(2,73) = 2.773, *P* = 0.069, *partial*η*^2^* = 0.071). The participants had a higher PSP score post-intervention than at baseline. There was a significant interaction between group and time (*F*(2,73) = 11.065, *P* < 0.001, *partial*η*^2^* = 0.233). Further analyses revealed that both VR-SCIT (*P* = 0.001) and TR-SCIT (*P* = 0.005) groups had a significantly higher score than the waiting-list group post-intervention, but there was no significant difference between groups at baseline (all *P*≈1.000).

## Discussion

In the present study, we developed a VR-SCIT with a gamification-oriented design according to the principles of TR-SCIT. Our primary findings indicate that VR-SCIT had a higher treatment adherence than TR-SCIT and a comparable efficacy.

Patients in the VR-SCIT group had a lower dropout rate than those in the TR-SCIT group at both week-3 and week-5 time-point. Notably, none of the participants voluntarily withdrew from the VR-SCIT sessions. These findings, as expected, indicate that VR-SCIT has a high acceptability among patients with schizophrenia. We cautiously speculate that the high compliance in VR-SCIT could be partly explained by its gamification-oriented design. Recently, gamification has been widely used in the research realm of psychosocial interventions for psychosis (31, 48). In gamification, the introduction of game elements can significantly compensate for patients’ lack of motivation toward the treatment (30, 31). However, our results revealed that six patients voluntarily withdrew from TR-SCIT. These findings are contrary to those from our previous work in community settings [Wang et al. (15)], in which we found that no participants withdrew from the intervention. We speculate that this inconsistency could be accounted for by the type of sample enrolled in these studies. In the study by Wang et al. (15), patients were recruited from Chinese community settings and received regular follow-up in accordance with the local mental health policy. However, in the present study, all the included patients were inpatients. The average length of hospital stay was approximately 30 days. In this sense, it was difficult to ensure the participants’ retention under the TR-SCIT program, which lasted for 5 weeks. Taken together, we could expect that the 3-week VR-SCIT program with a gamification-oriented design would be more feasible in different clinical settings.

Regarding efficacy, both the VR-SCIT and TR-SCIT demonstrated statistically significant improvements in the domains of emotion perception, metacognition, hostile attributional bias, and social functioning. These findings are mostly consistent with those of previous studies and support the efficacy of SCIT for the improvement of social cognition and social functioning in patients with schizophrenia. However, neither SCIT format showed an advantage over the waiting list group in improving the theory of mind. The lack of an effect on theory of mind is inconsistent with our previous work but consistent with other studies (10, 13). One explanation, as Roberts pointed out, may be that SCIT has less impact on higher-functioning patients (13). In the present study, the patients were hospitalized with acute episodes, many of which were first episodes. In contrast, in our previous study (15), participants were patients with chronic schizophrenia. In addition, we found that VR-SCIT showed an advantage over TR-SCIT in improving emotion perception and metacognition. We speculate that this may be associated with the more intense and immersive training in VR-SCIT than in TR-SCIT. In addition, the gamification design offered participants the opportunity to practice these skills.

In general, the present study was preliminary and had a few limitations. First, the sample size was small, which limits the generalizability of our findings. Second, the assessments of psychopathology and neuropsychological were only administered at baseline; thus, no conclusions can be drawn regarding whether VR-SCIT may be also effective for the improvement of psychotic symptoms or neurocognitive. Third, follow-up was not conducted after the intervention. Therefore, it is uncertain whether the observed efficacy was stable over time. Further multi-center, large-sample, long-term follow-up randomized controlled studies are needed in the future. In addition, our team is developing a VR-based social cognitive assessment tool to improve the ecological validity of the assessment.

In summary, despite several limitations, the present study provides the first evidence that VR-SCIT has the potential to improve social cognition in patients with schizophrenia. Although preliminary, it is suggested that the SCIT program, including the VR-based format, should become part of routine clinical interventions for patients with schizophrenia.

## Data availability statement

The raw data supporting the conclusions of this article will be made available by the authors, without undue reservation.

## Ethics statement

The studies involving human participants were reviewed and approved by the local ethics committee of the Seventh Hospital of Hangzhou. The patients/participants provided their written informed consent to participate in this study. Written informed consent was also obtained from the individuals for the publication of any potentially identifiable images or data included in this article.

## Author contributions

Z-HS contributed to data collection, data analyses, writing—original draft, and manuscript redaction and revisions. M-HL, YW, and Q-QL contributed to data collection and data analyses. Y-GW contributed to study design and manuscript redaction and revisions. All authors contributed to the article and approved the submitted version.
